# Laparoscopic Removal of Pelvic Hydatid Cysts in Young Female: A Case Report

**DOI:** 10.1155/2011/346828

**Published:** 2011-01-23

**Authors:** Kedar Gorad, Neeraj Rayate, Kunal Oswal, Ashish Krishna, Avanish Deshmukh, Sarvana Rajmanickam, Shailesh Puntambekar

**Affiliations:** Department of Minimal Access Cancer Surgery, Galaxy CARE Laparoscopy Institute, 25-A, Karve Road, Erandwane, Pune-411004, India

## Abstract

Hydatid disease is a zoonotic infection caused by larval stages of dog tapeworms belonging to the genus Echinococcus (family taeniidae) and is also referred to as echinococcosis. Human cystic echinococcosis caused by *E. granulosus* is the most common presentation and probably accounts for more than 95% of the estimated 2-3 million annual worldwide cases. The liver (70–80%) and lungs (15–25%) are the most frequent locations for echinococcal cysts. The diagnosis is made through the combined assessment of clinical, radiological, and laboratory findings. The treatment is mainly surgical, and, with appropriate diagnosis and treatment, prognosis is good. With advances and increasing experience in laparoscopic surgery, many more attempts have been made to offer the advantage of such a procedure to these patients (Chowbey et al. (2003)).

## 1. Case Presentation

**Figure 1:**
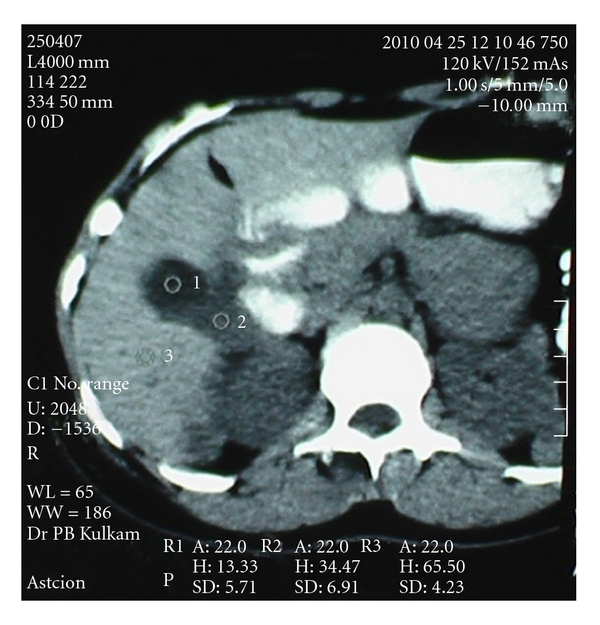
CT Scan of liver hydatid cysts.

**Figure 2:**
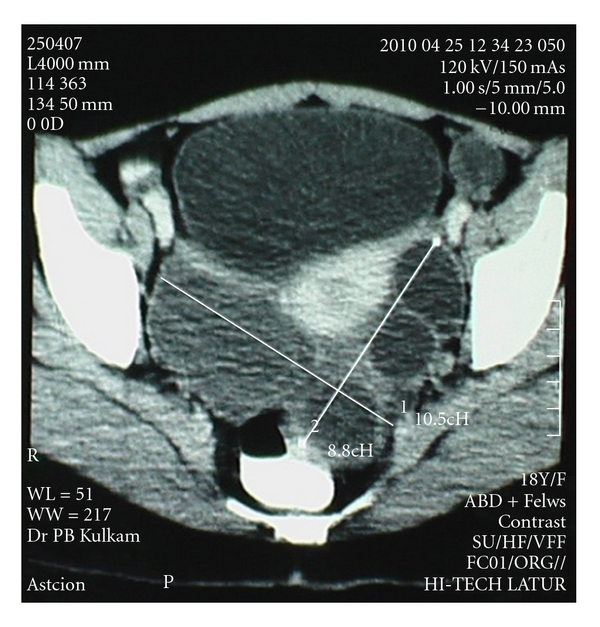
CT Scan showing hydatid cysts in the pelvis.

An 18-year-old girl presented with abdominal pain and nausea since seven months. She had undergone surgery for hydatid cyst in liver nine months before. On abdominal examination, tender firm masses were palpable in right hypochondrium and in pelvis, respectively, with limited mobility. Laboratory investigations were normal except marked eosiniphilia. USG abdomen revealed a space-occupying lesion in the pelvis and hydatid cysts in liver. CT scan of abdomen revealed two 7 × 5 and 4.5 × 4 sized cysts in liver (Figure 1). A separate cyst of 10.5 × 8.8 cm dimension was noted in the pelvis (Figure 2).

**Figure 3:**
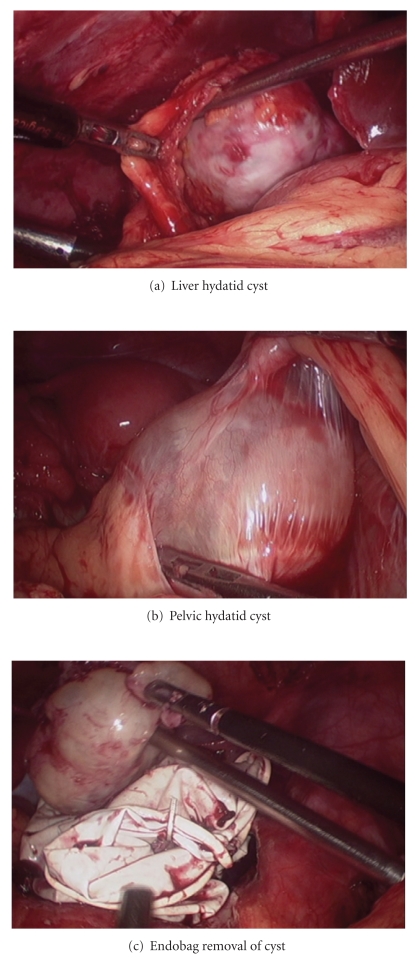
Intraoperative pictures.

Preoperatively albendazole was started to patient. Intraoperatively, prophylactic steroids were given to take care of some spillage. Hypertonic saline-soaked drapes and pads were used. During laparoscopy peripheral and cyst at porta were dissected and removed. Surprisingly there were two large and 5 small cysts in the pelvis, which were removed in toto without rupture (Figure 3). Patient had uneventful recovery. Followup USG abdomen after 6 months was within normal limit.

## 2. Discussion

Intraperitoneal hydatid cysts usually develop secondary to spontaneous or iatrogenic rupture of hepatic, splenic, or mesenteric cysts. Rarely isolated primary cyst may develop in the peritoneum without evidence of cysts in other intra-abdominal organs.

Primary peritoneal echinococcosis accounts for 2% of all abdominal hydatidosis [1]. Diagnosis is confirmed by eosinophilia and radio-imaging studies (abdominal sonography and computerized tomography). Primary peritoneal hydatid cyst presenting as ovarian, mesenteric, duplication and other intra-abdominal cysts have been reported. All these patients had evidence of hydatosis in other peritoneal organs [2]. Preoperative courses of Albendazole should be considered in order to sterilize the cyst, decrease the chance of anaphylaxis, decrease the tension in the cyst wall (thus reducing the risk of spillage during surgery), and reduce the recurrence rate postoperatively [3]. Intra-operatively, the use of hypertonic saline or 0.5% silver nitrate solutions before opening the cavities tends to kill the daughter cysts and therefore prevent further spread or anaphylactic reaction [3, 4].

Contamination of the peritoneal cavity was further prevented by extraction of cysts in an endobag. 

In our case, the liver cysts may be recurrence or new development, and peritoneal cysts were the consequence of previous surgery.

A recurrence rate of 2% [4] and survival rate of 95% have been reported in patient undergoing operative intervention [4]. The efficacy of Albendazole, as sole medical therapy, results in successful treatment in up to 40% of cases [3, 4].

## 3. Conclusion

We report a case of hydatid cysts within the pelvis due to previous peritoneal contamination which is of rare occurrence. Recurrence in liver is common but multiple cysts in pelvis are rare. Successful treatment without recurrence by laparoscopy was done.
